# *In Vitro* Activity of Meropenem-Vaborbactam against Clinical Isolates of KPC-Positive Enterobacteriaceae

**DOI:** 10.1128/AAC.01904-17

**Published:** 2017-12-21

**Authors:** Meredith A. Hackel, Olga Lomovskaya, Michael N. Dudley, James A. Karlowsky, Daniel F. Sahm

**Affiliations:** a International Health Management Associates, Inc., Schaumburg, Illinois, USA; b The Medicines Company, San Diego, California, USA; c Department of Medical Microbiology and Infectious Diseases, College of Medicine, University of Manitoba, Winnipeg, Manitoba, Canada

**Keywords:** meropenem, vaborbactam, KPC, carbapenemase, Enterobacteriaceae, Vabomere

## Abstract

Vaborbactam (formerly RPX7009) is a novel inhibitor of serine β-lactamases, including Ambler class A carbapenemases, such as KPCs. The current study evaluated the *in vitro* activity of the combination agent meropenem-vaborbactam against a global collection of 991 isolates of KPC-positive Enterobacteriaceae collected in 2014 and 2015 using the Clinical and Laboratory Standards Institute (CLSI) standard broth microdilution method. The MIC_90_ of meropenem (when tested with a fixed concentration of 8 μg/ml of vaborbactam) for isolates of KPC-positive Enterobacteriaceae was 1 μg/ml, and MIC values ranged from ≤0.03 to >32 μg/ml; 99.0% (981/991) of isolates had meropenem-vaborbactam MICs of ≤4 μg/ml, the U.S. FDA-approved MIC breakpoint for susceptibility to meropenem-vaborbactam (Vabomere). Vaborbactam lowered the meropenem MIC_50_ from 32 to 0.06 μg/ml and the MIC_90_ from >32 to 1 μg/ml. There were no differences in the activity of meropenem-vaborbactam when the isolates were stratified by KPC variant type. We conclude that meropenem-vaborbactam demonstrates potent *in vitro* activity against a worldwide collection of clinical isolates of KPC-positive Enterobacteriaceae collected in 2014 and 2015.

## INTRODUCTION

Carbapenem resistance has emerged worldwide in clinical isolates of Enterobacteriaceae (1–3). The spread of carbapenem-resistant Enterobacteriaceae, facilitated by either the horizontal spread of carbapenemase genes or the clonal expansion of carbapenem-resistant isolates (e.g., Klebsiella pneumoniae sequence type 258), has been identified to be a global public health threat and is of particular concern for patients afflicted with health care-associated infections (4, 5). Factors associated with escalating carbapenem resistance rates include the increased reliance on (and selective pressure from) carbapenems as treatment for the burgeoning number of infections caused by extended-spectrum β-lactamase (ESBL)-positive Enterobacteriaceae that have occurred worldwide over the last 2 decades as well as substandard infection control practices and the absence of antimicrobial stewardship programs in many hospitals (6, 7). The spread of carbapenemase genes both to colonizing flora and to potential pathogens is of particular concern because carbapenemases frequently confer resistance to all β-lactams, the most widely prescribed class of antimicrobial agents (8). The majority of carbapenemase-producing Enterobacteriaceae are also multidrug resistant (1, 2), limiting the treatment options available for empirical and directed therapy (9). The list of antimicrobial agents currently available to treat patients infected with carbapenem-resistant Gram-negative bacilli is short (aminoglycosides, tigecycline, colistin) and includes agents commonly associated with significant toxicities and increasing resistance or agents to which some species of Enterobacteriaceae show intrinsic resistance (6, 7, 10). New antimicrobial agents are urgently needed to address the increasing prevalence of carbapenem-resistant Enterobacteriaceae (4, 5).

Carbapenemases are classified into three of the four Ambler (molecular) classes of β-lactamase enzymes: class A (e.g., KPC), class B (e.g., NDM, VIM, IMP), and class D (e.g., OXA-48) enzymes. Class A, class C (AmpC), and class D β-lactamase have serine-based active sites. Class B enzymes have zinc-based active sites and are known as metallo-β-lactamases (MBLs). AmpC β-lactamases and ESBLs (a subset of class A β-lactamases) may also confer carbapenem resistance to isolates of Gram-negative bacilli when combined with porin mutations/loss, expression of efflux pumps, and/or alterations in penicillin-binding proteins (1–3, 6, 7, 9). KPCs have the greatest global distribution of all carbapenemases associated with Enterobacteriaceae (1–3, 7, 9). KPC-positive isolates are the most common carbapenemase-producing Enterobacteriaceae in the United States and have also been reported to be widespread in South and Central America, the Middle East, and China (7). In Europe, the highest incidences of KPC-positive Enterobacteriaceae are found in Italy and Greece (1–3, 7, 9).

A proven strategy to overcome β-lactamase-driven resistance is to restore the activity of an inactivated β-lactam agent by combining it with an inhibitor of the β-lactamase(s) responsible for the degradation of that β-lactam. Vaborbactam (formerly RPX7009) was developed specifically to inactivate KPC β-lactamases (11). It is a novel (first-in-class), non-β-lactam, cyclic boronic acid pharmacophore that inhibits serine β-lactamases of class A and class C, including KPC, IMI, SME, NMC-A, BKC-1, and FR-1 carbapenemases (8, 11–13), with no inhibition of mammalian serine proteases (11). Vaborbactam was optimized to be a potent inhibitor of serine β-lactamases using *in silico* modeling of the active sites of key serine β-lactamases, principally, KPCs (11). Vaborbactam possesses no antibacterial activity alone (MIC, >64 μg/ml) (12, 14). Mechanistically, the affinity of boronates, such as vaborbactam, for serine-based active sites of β-lactamases is due to the formation of a covalent complex between the catalytic serine side chain and the boronate moiety, which mimics the tetrahedral transition state of the acylation or deacylation reaction complex (11). Vaborbactam is structurally distinct from other new β-lactamase inhibitors, such as avibactam and relebactam, which are diazabicyclooctane inhibitors and also inhibit KPCs (1, 2). Avibactam is approved for use in combination with ceftazidime and is in clinical development in combination with other β-lactams, and relebactam is part of a combination with imipenem. KPCs are poorly inhibited by clavulanate, tazobactam, and sulbactam; and β-lactam–β-lactamase inhibitor combinations including these three older β-lactamase inhibitors have no utility in the treatment of infections due to carbapenem-resistant Enterobacteriaceae.

Meropenem-vaborbactam (Vabomere) in a fixed-dose combination of meropenem and vaborbactam has recently been approved by the U.S. FDA for the treatment of complicated urinary tract infections and acute pyelonephritis. The New Drug Application (NDA) included a phase 3 clinical trial to evaluate its efficacy in the treatment of complicated urinary tract infection, including acute pyelonephritis, in comparison to that of piperacillin-tazobactam (TANGO I; ClinicalTrials.gov identifier NCT02166476). A second phase 3 clinical trial in which meropenem-vaborbactam is being assessed for its efficacy for the treatment of serious infections due to carbapenem-resistant Enterobacteriaceae, including hospital-acquired and ventilator-associated pneumonia, in comparison to that of the best available antimicrobial therapy (TANGO II; ClinicalTrials.gov identifier NCT02168946) was ongoing at the time of NDA submission and review.

The current study evaluated the *in vitro* activities of meropenem-vaborbactam and seven comparator agents against a recent global collection of 991 clinical isolates of KPC-positive (OXA-48-negative and MBL-negative) Enterobacteriaceae collected in 2014 and 2015.

## RESULTS

Table 1 depicts the *in vitro* activities of meropenem-vaborbactam and its comparators against 991 clinical isolates of Enterobacteriaceae known to be KPC positive (and negative for genes for both OXA-48 and MBLs). The MIC_50_s and MIC_90_s of meropenem-vaborbactam for all isolates of Enterobacteriaceae tested were 0.06 and 1 μg/ml, respectively. These concentrations were 512-fold and >64-fold lower, respectively, than the MIC_50_ (32 μg/ml) and MIC_90_ (>32 μg/ml) of meropenem alone. The modal MIC of meropenem for all 991 isolates of Enterobacteriaceae also fell in the range of from >32 to ≤0.03 μg/ml in the presence of vaborbactam (Table 2). The MIC_50_ and MIC_90_ values of meropenem-vaborbactam for K. pneumoniae (MIC_50_, 0.12 μg/ml; MIC_90_, 1 μg/ml) were >4-fold higher and 4 to >32-fold higher, respectively, than the MIC_50_ and MIC_90_ values for Escherichia coli, Enterobacter spp., Klebsiella oxytoca, and Citrobacter spp. (Table 2). The MIC_90_ value of meropenem-vaborbactam for Serratia marcescens (1 μg/ml) was identical to that of K. pneumoniae. Reductions in MIC_50_ and MIC_90_ values similar to those demonstrated for meropenem-vaborbactam and meropenem alone were also observed when the values for ceftazidime-avibactam were compared with those for ceftazidime alone (Table 1). The MIC distributions of meropenem-vaborbactam for K. pneumoniae and S. marcescens were demonstrated to be much broader than those for the other species of Enterobacteriaceae tested (Table 2). Meropenem-vaborbactam (MIC_90_, 1 μg/ml) was more potent than all other agents tested, including tigecycline (MIC_90_, 2 μg/ml), ceftazidime-avibactam (MIC_90_, 4 μg/ml), and polymyxin B (MIC_90_, 16 μg/ml) (Table 1).

**TABLE 1:** *In vitro* activities of meropenem-vaborbactam and comparator agents against 991 clinical isolates of KPC-positive Enterobacteriaceae

Family, genus, or species^*a*^ (no. of isolates)	Antimicrobial agent(s)	MIC^*b*^ (μg/ml)			% of isolates with the following MIC interpretation^*c*^:		
		Range	50%	90%	Susceptible	Intermediate	Resistant
All Enterobacteriaceae^*d*^ (991)	Meropenem-vaborbactam	≤0.03 to >32	0.06	1	99.0	0.6	0.4
	Meropenem	2 to >32	32	>32	0	4.1	95.9
	Ceftazidime-avibactam	≤0.06 to >64	1	4	98.2		1.8
	Ceftazidime	1 to >64	>64	>64	3.0	2.5	94.5
	Tigecycline	≤0.06 to 8	1	2	95.8	3.6	0.6
	Minocycline	0.5 to >64	8	32	44.5	30.4	25.1
	Gentamicin	≤0.06 to >64	1	>64	63.4	6.3	30.4
	Polymyxin B	0.25 to >16	0.5	16	NA	NA	NA
K. pneumoniae (878)	Meropenem-vaborbactam	≤0.03 to >32	0.12	1	98.9	0.7	0.5
	Meropenem	2 to >32	>32	>32	0	1.9	98.1
	Ceftazidime-avibactam	≤0.06 to >64	1	4	98.2		1.8
	Ceftazidime	1 to >64	>64	>64	1.6	2.0	96.4
	Tigecycline	0.12 to 8	1	2	95.9	3.4	0.7
	Minocycline	1 to >64	8	32	44.2	32.0	23.8
	Gentamicin	≤0.06 to >64	1	>64	64.7	6.1	29.2
	Polymyxin B	0.25 to >16	0.5	16	NA	NA	NA
E. coli (35)	Meropenem-vaborbactam	≤0.03 to 0.12	≤0.03	≤0.03	100	0	0
	Meropenem	2 to 32	4	16	0	25.7	74.3
	Ceftazidime-avibactam	≤0.06 to 1	0.5	1	100		0
	Ceftazidime	2 to >64	64	>64	8.6	5.7	85.7
	Tigecycline	≤0.06 to 1	0.25	0.5	100	0	0
	Minocycline	0.5 to >64	4	32	51.4	11.4	37.1
	Gentamicin	0.25 to >64	1	>64	57.1	5.7	37.1
	Polymyxin B	0.25 to 1	0.5	0.5	NA	NA	NA
Enterobacter spp.^*e*^ (29)	Meropenem-vaborbactam	≤0.03 to 0.12	≤0.03	0.12	100	0	0
	Meropenem	2 to >32	8	>32	0	27.6	72.4
	Ceftazidime-avibactam	0.25 to 2	1	2	100		0
	Ceftazidime	4 to >64	32	>64	3.5	6.9	89.7
	Tigecycline	0.25 to 4	1	2	93.1	6.9	0
	Minocycline	1 to >64	16	64	41.4	3.5	55.2
	Gentamicin	0.25 to >64	4	>64	51.7	13.8	34.5
	Polymyxin B	0.25 to 16	0.5	1	NA	NA	NA
K. oxytoca (19)	Meropenem-vaborbactam	≤0.03 to 0.25	≤0.03	0.25	100	0	0
	Meropenem	2 to >32	4	32	0	21.1	78.9
	Ceftazidime-avibactam	≤0.06 to 16	0.5	4	94.7		5.3
	Ceftazidime	4 to >64	64	>64	15.8	5.3	78.9
	Tigecycline	0.12 to 2	0.5	2	100	0	0
	Minocycline	1 to >64	4	>64	52.6	36.9	10.5
	Gentamicin	0.25 to >64	8	>64	42.1	10.5	47.4
	Polymyxin B	0.5 to 1	0.5	0.5	NA	NA	NA
S. marcescens (16)	Meropenem-vaborbactam	≤0.03 to 2	0.06	1	100	0	0
	Meropenem	2 to >32	16	>32	0	6.2	93.8
	Ceftazidime-avibactam	≤0.06 to 32	0.5	2	93.8		6.2
	Ceftazidime	2 to >64	8	>64	37.5	12.5	50.0
	Tigecycline	0.5 to 4	1	4	81.3	18.7	0
	Minocycline	2 to 32	4	16	50.0	31.2	18.8
	Gentamicin	0.5 to >64	1	>64	68.8	0	31.2
	Polymyxin B	4 to >16	>16	>16	NA	NA	NA
Citrobacter spp.^*f*^ (13)	Meropenem-vaborbactam	≤0.03 to 0.12	≤0.03	0.06	100	0	0
	Meropenem	2 to 32	4	8	0	15.4	84.6
	Ceftazidime-avibactam	0.12 to 2	0.5	2	100		0
	Ceftazidime	4 to >64	64	>64	23.1	0	76.9
	Tigecycline	0.25 to 4	0.5	2	92.3	7.7	0
	Minocycline	1 to >64	8	>64	30.8	23.1	46.1
	Gentamicin	0.25 to >64	16	>64	46.2	0	53.8
	Polymyxin B	0.25 to 1	0.5	0.5	NA	NA	NA

a Species of Enterobacteriaceae with <10 isolates were grouped together with other species in their genus, and data are presented as data for the genus only.

b MIC_50_ and MIC_90_ values for individual genus or species were calculated when 10 or more isolates were tested.

c NA, not available. There are no CLSI, EUCAST, or U.S. FDA MIC breakpoints published for polymyxin B. Current CLSI MIC interpretative breakpoints were used for meropenem (susceptible, ≤1 μg/ml; intermediate, 2 μg/ml; resistant, ≥4 μg/ml), ceftazidime (susceptible, ≤4 μg/ml; intermediate, 8 μg/ml; resistant, ≥16 μg/ml), minocycline, and gentamicin. Current U.S. FDA MIC interpretative breakpoints were used for meropenem-vaborbactam (susceptible, ≤4 μg/ml; intermediate, 8 μg/ml; resistant, ≥16 μg/ml), ceftazidime-avibactam (susceptible, ≤8 μg/ml; resistant, ≥16 μg/ml), and tigecycline (susceptible, ≤2 μg/ml; intermediate, 4 μg/ml; resistant, ≥8 μg/ml). Vaborbactam was tested at a final concentration of 8 μg/ml.

d There was one isolate of Raoultella ornithinolytica in the study. It was included in the data set for all Enterobacteriaceae but not in a genus-specific subset of isolates. The MICs of meropenem and meropenem-vaborbactam for this isolate were >32 and 0.25 μg/ml, respectively.

e The 29 isolates of Enterobacter spp. comprised 17 Enterobacter cloacae isolates, 8 Enterobacter aerogenes isolates, 3 Enterobacter asburiae isolates, and 1 Enterobacter hormaechi isolate.

f The 13 isolates of Citrobacter spp. comprised 11 Citrobacter freundii isolates and 2 Citrobacter koseri isolates.

**TABLE 2:** Cumulative MIC distributions for meropenem-vaborbactam and meropenem against 991 clinical isolates of KPC-positive Enterobacteriaceae

Family, genus, species (no. of isolates)	Antimicrobial agent(s)	No. of isolates (cumulative % of isolates) inhibited at MIC (μg/ml)^*a*^ of:											
		≤0.03	0.06	0.12	0.25	0.5	1	2	4	8	16	32	>32
All Enterobacteriaceae^*b*^ (991)	Meropenem-vaborbactam	460 (46.4)	55 (52.0)	58 (57.8)	135 (71.4)	139 (85.5)	**80 (93.5)**	39 (97.5)	15 (99.0)	6 (99.6)	2 (99.8)	0 (99.8)	2 (100)
	Meropenem							41 (4.1)	78 (12.0)	125 (24.6)	148 (39.6)	140 (53.7)	**459 (100)**
K. pneumoniae (878)	Meropenem-vaborbactam	372 (42.4)	47 (47.7)	49 (53.3)	131 (68.2)	138 (83.9)	**78 (92.8)**	38 (97.2)	15 (98.9)	6 (99.5)	2 (99.8)	0 (99.8)	2 (100)
	Meropenem							17 (1.9)	46 (7.2)	104 (19.0)	130 (33.8)	130 (48.6)	451 (100)
E. coli (35)	Meropenem-vaborbactam	**34 (97.1)**	0 (97.1)	1 (100)									
	Meropenem							9 (25.7)	14 (65.7)	7 (85.7)	**3 (94.3)**	2 (100)	
Enterobacter spp. (29)	Meropenem-vaborbactam	22 (75.9)	3 (86.2)	**4 (100)**									
	Meropenem							8 (27.6)	5 (44.8)	3 (55.2)	9 (86.2)	1 (89.7)	**3 (100)**
K. oxytoca (19)	Meropenem-vaborbactam	17 (89.5)	0 (89.5)	0 (89.5)	**2 (100)**								
	Meropenem							4 (21.1)	7 (57.9)	5 (84.2)	1 (89.5)	**1 (94.7)**	1 (100)
S. marcescens (16)	Meropenem-vaborbactam	4 (25.0)	4 (50.0)	3 (68.8)	1 (75.0)	1 (81.3)	**2 (93.8)**	1 (100)					
	Meropenem							1 (6.3)	0 (6.3)	2 (18.8)	5 (0.50)	5 (81.3)	**3 (100)**
Citrobacter spp. (13)	Meropenem-vaborbactam	11 (84.6)	**1 (92.3)**	1 (100)									
	Meropenem							2 (15.4)	6 (61.5)	**4 (92.3)**	0 (92.3)	1 (100)	

a MIC_90_s are in boldface.

b There was one isolate of Raoultella ornithinolytica in the study. It was included in the data set for all Enterobacteriaceae but not in a genus-specific subset of isolates. The MICs of meropenem and meropenem-vaborbactam for this isolate were >32 and 0.25 μg/ml, respectively.

The percent susceptibility for all isolates of Enterobacteriaceae was greater for meropenem-vaborbactam (99.0% susceptible) than for ceftazidime-avibactam (98.2%) and tigecycline (95.8%). Of the 991 isolates of KPC-positive Enterobacteriaceae tested, 6 isolates (0.6%) tested as meropenem-vaborbactam intermediate, 4 isolates (0.4%) tested as meropenem-vaborbactam resistant, and 18 isolates (1.8%) tested as ceftazidime-avibactam resistant (Table 1). Of the 991 isolates tested, 5.3% (53/991) had a ceftazidime-avibactam MIC of 8 μg/ml (data not shown). The observed difference in percent nonsusceptibility between meropenem-vaborbactam (1.0% of isolates) and ceftazidime-avibactam (1.8% of isolates) was due to 14 of 18 ceftazidime-avibactam-resistant isolates (MIC, ≥16 μg/ml) testing as susceptible to meropenem-vaborbactam (MIC, ≤4 μg/ml) and 6 of 10 meropenem-vaborbactam-nonsusceptible isolates (MIC, ≥8 μg/ml) testing as susceptible to ceftazidime-avibactam (MIC, ≤8 μg/ml) (data not shown). In general, cross-resistance between meropenem-vaborbactam and ceftazidime-avibactam was uncommon, occurring in only 20.8% (5/24 isolates) of isolates resistant to either agent.

The MIC range for meropenem-vaborbactam for 991 isolates of KPC-positive Enterobacteriaceae was ≤0.03 to >32 μg/ml, with the MICs for only 2 isolates exceeding 16 μg/ml (both were K. pneumoniae isolates with MIC values of >32 μg/ml; 1 isolate was from Greece [and produced KPC-2], and the other isolate was from Italy [and produced KPC-3]). Of the two isolates with meropenem-vaborbactam MIC values of >32 μg/ml, one was resistant to ceftazidime-avibactam (MIC, 32 μg/ml) and had a polymyxin MIC of 0.5 μg/ml, whereas the other isolate was susceptible to ceftazidime-avibactam (MIC, 2 μg/ml) and had a polymyxin B MIC of >16 μg/ml.

The cumulative MIC distributions of meropenem-vaborbactam stratified by KPC variant are shown in Table 3. There were no appreciable differences in the activity of meropenem-vaborbactam against different KPC variants (KPC-2, KPC-3), suggesting that the differences in the activity of meropenem-vaborbactam observed between K. pneumoniae and S. marcescens and the other species of Enterobacteriaceae were due to factors other than the KPC variant present. Of the 18 ceftazidime-avibactam-resistant isolates (MIC, ≥16 μg/ml), 77.8% (14/18) produced KPC-3 (data not shown). A difference was observed between KPC variants with meropenem-vaborbactam MICs of ≥8 μg/ml (0.8% [5/610] of isolates producing KPC-2 and 1.3% [5/373] of isolates producing KPC-3) (Table 3) and those with ceftazidime-avibactam MICs of ≥16 μg/ml (0.7% [4/610] of isolates producing KPC-2 and 3.8% [14/373] of isolates producing KPC-3) (data not shown).

**TABLE 3:** Cumulative MIC distributions for meropenem-vaborbactam stratified by KPC variant

KPC variant (no. of isolates)	No. of isolates (cumulative % of isolates) inhibited at MIC (μg/ml)^*a*^ of:											
	≤0.03	0.06	0.12	0.25	0.5	1	2	4	8	16	32	>32
KPC-2 (610)	294 (48.2)	42 (55.1)	39 (61.4)	67 (72.5)	79 (85.4)	**46 (93.0)**	27 (97.4)	11 (99.2)	4 (99.8)	0 (99.8)	0 (99.8)	1 (100)
KPC-3 (373)	161 (43.2)	13 (46.6)	18 (51.5)	68 (69.7)	58 (85.3)	**34 (94.4)**	12 (97.6)	4 (98.7)	2 (99.2)	2 (99.7)	0 (99.7)	1 (100)
KPC-5 (2)	1 (50.0)	0 (50.0)	1 (100)									
KPC-6 (1)	1 (100)											
KPC-9 (2)	0 (0)	0 (0)	0 (0)	0 (0)	2 (100)							
KPC-18 (3)	3 (100)											
All isolates (991)	460 (46.4)	55 (52.0)	58 (57.8)	135 (71.4)	139 (85.5)	**80 (93.5)**	39 (97.5)	15 (99.0)	6 (99.6)	2 (99.8)	0 (99.8)	2 (100)

a MIC_90_s are in boldface when 10 or more isolates of a KPC variant were present.

The cumulative MIC distributions for meropenem-vaborbactam stratified by KPC, AmpC, and ESBL genotypes are shown in Table 4. There were no appreciable differences in the activity of meropenem-vaborbactam against isolates which coproduced AmpC enzymes or ESBLs and KPC (MIC_90_ values of 0.06 μg/ml and 1 μg/ml, respectively, compared to an MIC_90_ of 1 μg/ml for isolates which produced only KPC) (Table 4).

**TABLE 4:** Cumulative MIC distributions for meropenem-vaborbactam stratified by genotype

Genotype (no. of isolates)	No. of isolates (cumulative % of isolates) inhibited at MIC (μg/ml)^*a*^ of:										
	≤0.03	0.06	0.12	0.25	0.5	1	2	4	8	16	>32
KPC AmpC^*b*^ (34)	29 (78.4)	**2 (94.1)**	3 (100)								
KPC AmpC ESBL^*c*^ (8)	6 (75.0)	1 (87.5)	1 (100)								
KPC ESBL^*d*^ (346)	171 (49.4)	23 (56.1)	16 (60.7)	42 (72.8)	47 (86.4)	**27 (94.2)**	16 (98.8)	1 (99.1)	2 (99.7)	0 (0)	1 (100)
KPC only (603)	254 (42.1)	29 (46.9)	38 (53.2)	93 (68.7)	92 (83.9)	**53 (92.7)**	23 (96.5)	14 (98.8)	4 (99.5)	2 (99.8)	1 (100)

a MIC_90_s are is boldface when 10 or more isolates of a genotype were present.

b AmpC enzymes comprised ACT/MIR (*n* = 13 isolates) and CMY II (*n* = 21 isolates).

c AmpC enzymes comprised ACT/MIR (*n* = 2 isolates) and CMY II (*n* = 6 isolates); extended spectrum β-lactamase (ESBL) enzymes comprised CTX-M (*n* = 7 isolates) and SHV (*n* = 1 isolates).

d ESBL enzymes comprised CTX-M (*n* = 167 isolates), CTX-M and SHV (*n* = 9 isolates), and SHV (*n* = 170 isolates).

## DISCUSSION

In the current study, we observed that meropenem-vaborbactam inhibited 99.0% of KPC-positive isolates of Enterobacteriaceae at ≤4 μg/ml, the U.S. FDA MIC breakpoint for susceptibility (15). In the current study, the *in vitro* activity of meropenem-vaborbactam was equivalent to that of ceftazidime-avibactam (to which 98.2% of isolates were susceptible) and tigecycline (to which 95.8% of isolates were susceptible) (Table 1). Meropenem-vaborbactam was demonstrated to be a more potent antimicrobial agent *in vitro* than ceftazidime-avibactam, tigecycline, and all other antimicrobial agents tested against the recent worldwide collection of clinical isolates of KPC-positive Enterobacteriaceae tested (Table 1). On the basis of the MIC_90_s, meropenem-vaborbactam (MIC_90_, 1 μg/ml) was four times more potent than ceftazidime-avibactam and >64 times more potent than meropenem alone.

Four previous studies that determined the *in vitro* activity of meropenem-vaborbactam against clinical isolates of Gram-negative bacilli and in which subsets of isolates were phenotypically or molecularly characterized for ESBLs, carbapenemases, or other mechanisms of carbapenem resistance have been published (11, 14, 16, 17). Initially, Hecker et al. demonstrated that vaborbactam, at a fixed concentration of 4 μg/ml, reduced the MICs of biapenem, meropenem, ertapenem, and imipenem by ≥64-, ≥32-, ≥16-, and ≥32-fold, respectively, when tested against KPC-positive E. coli, Enterobacter cloacae, and Klebsiella isolates (11). These investigators also reported that vaborbactam potentiated the activity of cefepime against isolates producing class A and D β-lactamases with extended-spectrum activity against cephalosporins (CTX-M, SHV, TEM, OXA-2, OXA-1/OXA-30) and isolates with chromosomally encoded or transferable AmpC β-lactamases (11).

Lapuebla et al. tested meropenem in combination with vaborbactam (8 μg/ml) against a panel of 121 carbapenem-resistant KPC-positive isolates of K. pneumoniae and reported that the MIC_50_, MIC_90_, and MIC range were 0.03, 0.5, and ≤0.004 to >64 μg/ml, respectively, and that 98.5% (131/133) of KPC-positive Enterobacteriaceae (including 5 isolates of E. coli and 7 isolates of Enterobacter spp.) were inhibited by meropenem-vaborbactam at a concentration of 1 μg/ml (16). Lapuebla et al. also observed that vaborbactam had little to no effect on meropenem MICs for meropenem-nonsusceptible Acinetobacter baumannii isolates containing OXA-type carbapenemases or for Pseudomonas aeruginosa isolates (16). In addition, Lapuebla et al. reported that meropenem-vaborbactam MICs were 8- to 16-fold higher for isolates with diminished *ompK35* and *ompK36* expression than for isolates producing the same β-lactamases without permeability changes (16).

Castanheira et al. evaluated the activity of meropenem-vaborbactam against 315 serine carbapenemase-producing Enterobacteriaceae isolates, including 308 KPC-positive isolates, using checkerboard-designed panels and reported a maximum potentiation for vaborbactam activity at a concentration of 8 μg/ml (14). Castanheira et al. also reported that 93.7% of the 315 serine carbapenemase-producing isolates of Enterobacteriaceae were inhibited at a meropenem MIC of ≤1 μg/ml (vaborbactam concentration, 8 μg/ml) and that 96.5% of isolates were inhibited at a meropenem concentration of ≤2 μg/ml (14). The MIC_50_ and MIC_90_ for the 315 isolates were ≤0.06 and 1 μg/ml, respectively (14). These investigators identified seven isolates with meropenem-vaborbactam MICs of ≥16 μg/ml. All seven isolates were K. pneumoniae, four of which coproduced an MBL (MIC, 16 to >64 μg/ml); the other three isolates demonstrated decreased expression of *ompK37* and/or elevated expression of the AcrAB-TolC efflux system (MIC, 16 μg/ml) (14). Earlier, Livermore and Mushtaq also reported that an outer membrane porin deficiency combined with the presence of β-lactamases can diminish the effect of vaborbactam combined with biapenem, an observation that suggested that the utility of a carbapenem–β-lactamase inhibitor combination against certain isolates may be limited (12). Livermore and Mushtaq tested vaborbactam in combination with biapenem against 300 Enterobacteriaceae isolates, including isolates carrying KPC-type enzymes or another class A serine β-lactamase alone or in combination with an ESBL, derepressed AmpC, or an intrinsic resistance mechanism (12). These investigators determined that vaborbactam potentiated the activity of biapenem against KPC-positive isolates; however, the activity of biapenem-vaborbactam against isolates producing class B or D (OXA-48) β-lactamases was limited (12).

Most recently, Castanheira and coworkers studied >10,000 clinical isolates of Enterobacteriaceae collected worldwide in 2014 and reported MIC_90_s of meropenem-vaborbactam of 0.06, 32, 0.5, >32, and 1 μg/ml (vaborbactam was tested at a fixed concentration of 8 μg/ml) for all isolates, carbapenem-resistant Enterobacteriaceae, KPC producers, non-KPC-producing carbapenem-resistant Enterobacteriaceae isolates, and multidrug-resistant isolates, respectively (17). Overall, meropenem-vaborbactam inhibited 99.1% of Enterobacteriaceae at a meropenem MIC of ≤1 μg/ml (17). All but 5 of 135 (3.7%) KPC-producing isolates were inhibited by meropenem-vaborbactam at 1 μg/ml, a rate slightly lower than that observed in the current study (6.5%) (Table 3). All KPC-producing isolates in the study by Castanheira et al. were inhibited by meropenem-vaborbactam at an MIC of 8 μg/ml (17).

Previously, vaborbactam was also demonstrated to possess pharmacokinetics similar to those of β-lactam agents, including carbapenems, and displayed efficacy as a treatment for infections caused by KPC-positive isolates of Escherichia coli, Enterobacter cloacae, and K. pneumoniae in a neutropenic mouse thigh infection model (11, 18, 19). Meropenem-vaborbactam has also shown activity in an *in vitro* hollow-fiber model that simulated human exposure, where the data generated support for the use of meropenem-vaborbactam (vaborbactam concentration, 8 μg/ml) for the treatment of infections caused by KPC-positive carbapenem-resistant Enterobacteriaceae isolates with meropenem MICs as high as 8 μg/ml (20).

In the current study, the difference between the KPC variants associated with meropenem-vaborbactam MICs of ≥2 μg/ml (7.0% [43/610 isolates] for isolates producing KPC-2 and 5.6% [21/373 isolates] for isolates producing KPC-3) and meropenem-vaborbactam MICs of ≥4 μg/ml (2.6% [16/610 isolates] for isolates producing KPC-2 and 2.4% [9/373 isolates] for isolates producing KPC-3) (Table 3) and those associated with ceftazidime-avibactam MICs of ≥16 μg/ml (0.7% [4/610 isolates] for isolates producing KPC-2 and 3.8% [14/373 isolates] for isolates producing KPC-3) is noteworthy. Other authors have reported higher MICs of ceftazidime-avibactam for isolates carrying KPC-3 than for those carrying KPC-2 (21) and the emergence of resistance to ceftazidime-avibactam due to plasmid-borne KPC-3 mutations during treatment of carbapenem-resistant K. pneumoniae infections (22). The differences observed between the subsets of isolates resistant to meropenem-vaborbactam and ceftazidime-avibactam may also be related to the observation that the inhibition of KPC-2 by vaborbactam does not involve S130, a residue important for inhibition by avibactam (R. Tsivkovski, M. Totrov, and O. Lomovskaya, presented at Microbe 2016, Boston, MA).

The intent of the current study was to add to the limited amount of available *in vitro* data on the meropenem-vaborbactam MICs for KPC-positive Enterobacteriaceae isolates (14, 16, 17). Our data align with the findings of these previous studies and show that vaborbactam restores the *in vitro* activity of meropenem against the majority of isolates of Enterobacteriaceae carrying KPCs that would otherwise be nonsusceptible to carbapenems. On the basis of the results of our current study, meropenem-vaborbactam appears to be a promising, novel carbapenem–β-lactamase inhibitor combination. Its continued clinical development may provide a valuable therapeutic option for treating infections caused by antimicrobial-resistant Gram-negative bacilli in the future.

## MATERIALS AND METHODS

### Bacterial isolates.

The isolates of KPC-positive Enterobacteriaceae tested in this study (*n* = 991) were randomly selected from isolates in the frozen stock culture collection of International Health Management Associates, Inc. (IHMA; Schaumburg, IL, USA), collected in 2014 and 2015 for a global clinical isolate surveillance study on the basis of their β-lactamase content and year of isolation. In total, 580 isolates were selected in 2014 and 411 isolates were selected in 2015. All 991 KPC-positive isolates were previously determined to be OXA-48 negative and MBL negative by a protocol that included screening of the isolates for the presence of genes encoding the following β-lactamases using published multiplex PCR assays as described previously (24): ESBLs (TEM, SHV, CTX-M, VEB, PER, GES), AmpC enzymes (ACC, ACT, CMY, DHA, FOX, MIR, MOX), and carbapenemases (KPC, OXA-48, GES, IMP, VIM, NDM, SPM, GIM). The detected β-lactamase genes were amplified using flanking primers and sequenced. Enzyme subtypes were determined by comparison against the subtypes in the database maintained by the National Center for Biotechnology Information (www.ncbi.nlm.nih.gov). Isolates positive for OXA-48 or an MBL were excluded from the current study because previous publications have documented that vaborbactam does not inhibit these enzymes (13).

The 991 isolates comprised 878 K. pneumoniae (88.6% of isolates), 35 E. coli (3.5%), 19 Klebsiella oxytoca (1.9%), 17 Enterobacter cloacae (1.7%), 16 Serratia marcescens (1.6%), 11 Citrobacter freundii (1.1%), 8 Enterobacter aerogenes (0.8%), 3 Enterobacter asburiae (0.3%), and 2 Citrobacter koseri (0.2%) isolates and one isolate each of Enterobacter hormaechi (0.1%) and Raoultella ornithinolytica (0.1%). Of the 991 isolates, 496 isolates were from Europe (12 countries; 242 isolates producing KPC-2, 252 isolates producing KPC-3, 2 isolates producing KPC-9), 371 were from Latin America (9 countries; 326 isolates producing KPC-2, 42 isolates producing KPC-3, 2 isolates producing KPC-5, 1 isolate producing KPC-6), 96 from North America (2 countries; 19 isolates producing KPC-2, 74 isolates producing KPC-3, 3 isolates producing KPC-18), 16 from the Asia-Pacific region (3 countries; all 16 isolates produced KPC-2), and 12 from the Middle East (1 country; 7 isolates producing KPC-2, 5 isolates producing KPC-3). All isolates were originally grown in clinical microbiology laboratories from specimens from patients with documented infection, with a limit of one isolate per patient. The identities of all isolates were determined by IHMA using matrix-assisted laser desorption ionization–time of flight mass spectrometry (Bruker Daltonics, Billerica, MA, USA).

### Antimicrobial susceptibility testing.

All aspects of antimicrobial susceptibility testing, including broth microdilution panel production, panel inoculation, incubation, MIC reading, and MIC interpretation, followed Clinical and Laboratory Standards Institute (CLSI) standard methods and were performed on-site at IHMA (10, 25). Broth microdilution panels included the following antimicrobial agents: meropenem-vaborbactam (doubling dilution range tested, 0.03/8 to 32/8 μg/ml), meropenem (0.03 to 32 μg/ml), ceftazidime-avibactam (0.06/4 to 64/4 μg/ml), ceftazidime (0.06 to 64 μg/ml), tigecycline (0.06 to 8 μg/ml), minocycline (0.03 to 64 μg/ml), gentamicin (0.06 to 64 μg/ml), and polymyxin B (0.12 to 16 μg/ml). Vaborbactam and avibactam were provided to IHMA by The Medicines Company (San Diego, CA). All other antimicrobial agents were purchased from the U.S. Pharmacopeia (Rockville, MD). The broth microdilution panels were incubated at 35°C for 16 to 20 h in ambient air before MIC endpoints were read. All compounds tested were dissolved according to CLSI specifications and then further diluted in cation-adjusted Mueller-Hinton broth (CAMHB) to generate the sequential dilutions required to produce the broth microdilution panels (25). Colonies were taken directly from a second-pass culture plate, and a suspension with a turbidity that was the equivalent to that of a 0.5 McFarland standard was prepared using normal saline. Inoculation of the MIC plates took place within 15 min of adjustment of the turbidity of the inoculum suspension. MICs were interpreted using current CLSI breakpoints (25), with the following exceptions: U.S. FDA guidelines were used to interpret the MICs of meropenem-vaborbactam (susceptible, ≤4 μg/ml; intermediate, 8 μg/ml; resistant, ≥16 μg/ml) (15), ceftazidime-avibactam (susceptible, ≤8 μg/ml; resistant, ≥16 μg/ml) (26), and tigecycline (susceptible, ≤2 μg/ml; intermediate, 4 μg/ml; resistant, ≥8 μg/ml) (27). Polymyxin B lacks CLSI, U.S. FDA, or European Committee on Antimicrobial Susceptibility Testing (EUCAST) breakpoints for Enterobacteriaceae. Quality control testing was performed on each day of testing using E. coli ATCC 25922, P. aeruginosa ATCC 27853, K. pneumoniae ATCC 700603, K. pneumoniae 1074, and K. pneumoniae BAA1705. All quality control results were within specified CLSI ranges (10).
